# Proof of concept and development of a couple-based machine learning model to stratify infertile patients with idiopathic infertility

**DOI:** 10.1038/s41598-021-03165-3

**Published:** 2021-12-14

**Authors:** Guillaume Bachelot, Rachel Lévy, Anne Bachelot, Céline Faure, Sébastien Czernichow, Charlotte Dupont, Antonin Lamazière, Alifert Collaborative Group, Isabelle Aknin, Isabelle Cedrin-Durnerin, Steven Cens, Nathalie di Clemente, Jean-Louis Guéant, Serge Hercberg, Yoann Lalatonne, Chrystèle Racine, Nathalie Sermondade, Angela Sutton, Claude Uthurriague, Jean-Philippe Wolf, Alain Favier

**Affiliations:** 1 Service de Biologie de La Reproduction-CECOS, Hôpital Tenon, AP-HP/Sorbonne Université, 75020 Paris, France; 2 Sorbonne Université, Saint Antoine Research Center, INSERM UMR 938, 75012 Paris, France; 3 Service d’Endocrinologie et Médecine de La Reproduction, Centre de Référence Des Maladies Endocriniennes Rares de La Croissance et du Développement, Centre Des Pathologies Gynécologiques Rares, Hôpital Pitié Salpêtrière (APHP), Sorbonne Université, 75013 Paris, France; 4 Université de Paris, INSERM, UMR1153, Epidemiology and Biostatistics Sorbonne Paris Cité Center (CRESS), METHODS team, Service de Nutrition, Hôpital Européen Georges Pompidou, AP-HP, Paris, France; 5 Département de Métabolomique Clinique, Hôpital Saint Antoine, AP-HP/Sorbonne Université, 27 Rue Chaligny, 75012 Paris, France; 6 Unité Fonctionnelle de Biologie de La Reproduction, Histologie—Embryologie—Cytogénétique, Hôpital Nord, Saint-Étienne, France; 7 Service de Médecine de La Reproduction, Hôpital Jean Verdier, APHP, Bondy, France; 8 Centre d’AMP de PAU, Polyclinique de Navarre, Pau, France; 9 Laboratoire de Biochimie, CHU Brabois de Nancy, Université de Lorraine, Lorraine, France; 10 EREN, INSERM U557; INRA; CNAM; Université Paris 13, CRNH IdF, 93017 Bobigny, France; 11 Laboratoire de Médecine Nucléaire, 125 Route de Stalingrad, 93009 Bobigny, France; 12 Laboratoire de Biochimie, Hôpital Jean Verdier, APHP, Paris, France; 13 Centre d’AMP de PAU, Polyclinique de Navarre, Pau, France; 14 Service d’Histologie-Embryologie-Biologie de La Reproduction, Hôpital Cochin, APHP, Paris, France; 15 Département de Biologie Intégrée, Grenoble Hospital, 38043 Grenoble Cedex 09, France

**Keywords:** Diagnostic markers, Machine learning, Reproductive signs and symptoms, Quality of life

## Abstract

We aimed to develop and evaluate a machine learning model that can stratify infertile/fertile couples on the basis of their bioclinical signature helping the management of couples with unexplained infertility. Fertile and infertile couples were recruited in the ALIFERT cross-sectional case–control multicentric study between September 2009 and December 2013 (NCT01093378). The study group consisted of 97 infertile couples presenting a primary idiopathic infertility (> 12 months) from 4 French infertility centers compared with 100 fertile couples (with a spontaneously conceived child (< 2 years of age) and with time to pregnancy < 12 months) recruited from the healthy population of the areas around the infertility centers. The study group is comprised of 2 independent sets: a development set (n = 136 from 3 centers) serving to train the model and a test set (n = 61 from 1 center) used to provide an unbiased validation of the model. Our results have shown that: (i) a couple-modeling approach was more discriminant than models in which men’s and women’s parameters are considered separately; (ii) the most discriminating variables were anthropometric, or related to the metabolic and oxidative status; (iii) a refined model capable to stratify fertile vs. infertile couples with accuracy 73.8% was proposed after the variables selection (from 80 to 13). These influential factors (anthropometric, antioxidative, and metabolic signatures) are all modifiable by the couple lifestyle. The model proposed takes place in the management of couples with idiopathic infertility, for whom the decision-making tools are scarce. Prospective interventional studies are now needed to validate the model clinical use.

Trial registration: NCT01093378 ALIFERT https://clinicaltrials.gov/ct2/show/NCT01093378?term=ALIFERT&rank=1. Registered: March 25, 2010.

## Introduction

The World Health Organization (WHO) recognizes infertility as a public health issue defined as the lack of pregnancy after > 12 months of regular unprotected sexual intercourse^1^. Infertility affects 12.5% of women and 10% of men^2^.

The diagnosis and infertility management are a painful and long journey, especially for idiopathic or unexplained infertility. The latter condition is defined by a lack of diagnosis for couples that have failed to conceive after one or two years of non-protected sexual intercourse^3^ and affects 30 to 40% of infertile couples^4^. Standard investigation involving the tests of ovulation, tubal patency, and semen analysis. In addition to generating anxiety in the couple, the diagnostic wavering of idiopathic infertility and empirical treatments represent a massive cost to health care systems^4^. Even if no cause is identified, environment and lifestyle have been suspected as potential idiopathic infertility causes^5^. For both men and women, overweight and metabolic syndrome are recognized to negatively affect fertility^6,7^, but also on achieving pregnancy after assisted reproductive technology (ART)^8–10^. Studies showed increased ovulation disorders and miscarriages in obese or overweight women^11,12^. Likewise, overweight or obesity in men affected sperm parameters but also sperm DNA fragmentation^13,14^.

Consequently, it is important to focus again on modifiable risk factors among infertile couples using the resources of statistical modeling.

In a previous study of idiopathic infertile couples and fertile couples (the ALIFERT clinical study, NCT01093378), we observed that the sedentary behavior, physical inactivity, body composition, and metabolic disorders are significant factors of idiopathic infertility among men and women^15–18^. In this previous ALIFERT study, parameters for men and women were considered independently in order to tackle specific infertility issues. However, the mechanisms leading to idiopathic infertility are likely complex and multifactorial and the couple man + woman is also considered presently.

In the recent years, alongside deep learning and neuronal networks, machine learning computational models such as the Bayesian network, random forest, and partial least square (PLS) have driven substantial advances in the integration and treatment of complex bio-clinical signatures^19,20^. In this study, we propose to evaluate the performance of a couple-based Orthogonal Partial Least Square (OPLS) model facilitating the medical decision-making process in the context of idiopathic infertility. Our hypothesis is such a multivariate model, in which the man + woman couple formed a single observation and, in which the biochemical, clinical, and anthropometric variables of both men and women are merged together can discriminate efficiently between fertility and idopiopathic infertily.

To our knowledge, this study in the field of idiopathic infertility classification is the first to evaluate comprehensively a machine-learning algorithm and to assess the generalizability of this model across various diagnostic centers. The proven possibility prompts for a future interventional study.

## Materials and methods

### Subjects

Fertile and infertile couples were recruited in the ALIFERT cross-sectional case–control study between September 2009 and December 2013^18^ (National biomedical research Id. P071224, ethics committee approval [“Comité de Protection des Personnes”] AOM 2009-A00256-51, NEudra CT 08180, clinicaltrials.gov NCT01093378). The clinical study was performed in accordance with relevant guidelines/regulations. Informed consent was obtained from all participants. The research involving human research participants was performed in accordance with the Declaration of Helsinki.

ALIFERT was designed as a multicentric study. The study group consisted of 97 infertile couples (presenting a primary idiopathic infertility > 12 months) recruited from four infertility centers in France (Hôpital Jean-Verdier ART center, Bondy (JV); Hôpital Nord ART center, Saint-Étienne (SE); Polyclinique de Navarre ART center, Pau (PAU); Cochin Hospital ART center, Paris (CCH)) and 100 fertile couples (with a spontaneously conceived child under 2 years of age with time to pregnancy less than 12 months) recruited from the general population in areas of the participating centers. Men were under 45 years of age and women under 38 years of age.

Infertile couples presented a infertility > 12 months, of which the primary idiopathic chateracter is based on the following investigations: Men did not present severe sperm alteration nor urogenital pathology, Women did present neither anovulation, ovarian failure on the basis of follicle count nor hormone balance at day 3 (FSH, LH, and estradiol) nor uterotubal pathology assessed by hysterosalpingography. Patients with current or previous proven metabolic or digestive disease were excluded.

The control group had a spontaneously conceived child under 2 years of age with time to pregnancy less than 12 months. Eligibility criteria have been detailed previously^18^.

The study group was comprised of two independent cohorts: a development set serving to train the model (n = 136), including 73 infertile and 63 fertile couples from JV, SE, and PAU, and a test set (n = 61), including 24 infertile and 37 fertile couples from CCH. The development set was used to tune the machine-learning model, and the external validation set was used to evaluate the model performance. Further model refinement was also conducted on the overall cohort (n = 196) couples.

### Input data acquisition

Written informed consent was collected for both fertile and infertile couples. To note, all variables collected from women were encoded with a prefix of “w” before the variable name (i.e. *w_variable*). Variables without prefix were collected from men.

#### Assessments

Study and control subjects have been assessed by the same trained investigators using the same calibrated devices.

#### Anthropometric assessments

The investigator measured height, weight (Tanita BC-420MA analyzer), and waist circumference measured at the narrowest point between the lower border of the ribs and the iliac crest.

#### Blood pressure assessment

Systolic and diastolic blood pressures have been measured using a sphygmomanometer cuff after 5 min rest around patient forearm in a supine position. The systolic and diastolic pressures were the mean of right and left values.

#### Carbon monoxide status

Exhaled carbon monoxide (CO) has been measured in parts per million (ppm), as a supportive indicator with the underlying assumption that exhaled CO in smokers^21^ is higher than in non-smokers. Exhaled CO measurement was performed by having subjects exhale completely then inhale fully in open air, withhold their breath for 10 s, and then exhale completely into the portable CO monitor (Tabataba analyser-FIM medical, Villeurbanne 69625 France)^21^.

#### Blood samples and analyses

Blood samples were collected after 12 h fasting for measurement of fresh plasma total cholesterol, high-density lipoprotein (HDL-cholesterol), low-density lipoprotein (LDL-cholesterol), triglycerides and glucose. Serum and plasma were stored at − 80 °C until further analyses.

#### LC–MS/MS steroid profiling

Steroid profiles were measured in serum by liquid chromatography–mass spectrometry (LC–MS/MS) as described elsewhere^22^.

#### Antioxidants and micronutrients

Serum vitamin D (ng/mL), vitamin B9 (folic acid-erytho) (nmol), vitamin B9 (nmol/L), vitamin B12 (pM), alpha-Tocopherol (vitamin E) (mmol/L), Zinc (mmol/L), Selenium (mmol/L), Vitamin C (mg/mL), alpha-Carotene (mmol/L), beta-carotene (mmol/L), Lycopene, Lutein, Zeaxanthin, beta-Kryptoxanthin and Retinol (vitamin A) were assayed at Département de Biologie intégrée – Biologie nutritionnelle et stress oxydatif (Grenoble hospital avenue du Maquis du Grésivaudan BP 217—38043 Grenoble Cedex 09).

Serum ascorbic acid (vitamin C) was determined by using an automated method based on the principle of continuous flow. Serum retinol, tocopherol and carotenoids (lutein, zeaxanthin, beta-cryptoxanthin, lycopene, alpha-carotene and beta-carotene) were measured by HPLC (Biotek-Kontron, Montigny-le-Bretonneux, France). Serum zinc concentrations were measured by using flame atomic absorption spectrometry (model 3110; Perkin Elmer, Norwalk, CT) and selenium by atomic absorption spectrometry (4100 ZL; Perkin Elmer)^23,24^.

In total 50 variables from men and 30 from women were collected and input in the models.

### Statistical analysis and data processing

The data sets were tested with Shapiro–Wilks in order to evaluate their distribution (Supplementary Tables 1a–1d)^25^. When the distribution was not normal, a non-parametric Wilcoxon–Mann–Whitney test was applied^26^. If the distribution was normal, a t-test was performed. These tests were performed using the Rstudio® software (version 3.6.1). The data were loaded into SIMCA 15® software (version 15, Umetrics, Västerbotten, Sweden) in order to perform the Principal Component Analysis (PCA) and Orthogonal Partial Least Square-Discriminant Analysis (OPLS-DA). (For more details see Supplementary material section).

We calculated the model starting from 50 variables in men, and 30 variables in women. Furtheron we have matched the 2 gender sets to generate a so-called “couple” matrix comprised of 80 variables. To our knowledge, the implementation of an algorithm taking into account the combined parameters of the matched man-woman couple has not been yet tested for infertility.

PCA, an unsupervised method allowing the data dimension reduction and the exclusion of outliers was applied to the development set. After the removal of 1 outlying couple the discriminant analysis OPLS-DA has served to calculate the regression of the multiple factors *versus* the couple fertility/intertility status. The supervised multivariate machine learning algorithm^27,28^, was trained using the development set (n = 136) , the model’s performance being evaluated using the external validation set (n = 31). Based on various studies, the OPLS approach is considered particularly appropriate to circumvent difficulties of multiple variables colinearity and missing data, and has already been used in healthcare^29–31^. Eventualy, we also trained and evaluated 4 other machine-learning models (Support vector machine, Nearest Neighbors Classifier, Decision Tree and logistic regression using Python 3.8.2, Scikit-Learn library 0.22.2, Numpy library 1.18.1, and Pandas library 1.0.1) to confirm the discriminative power of the data, independently of the chosen method.

**Figure 1:**
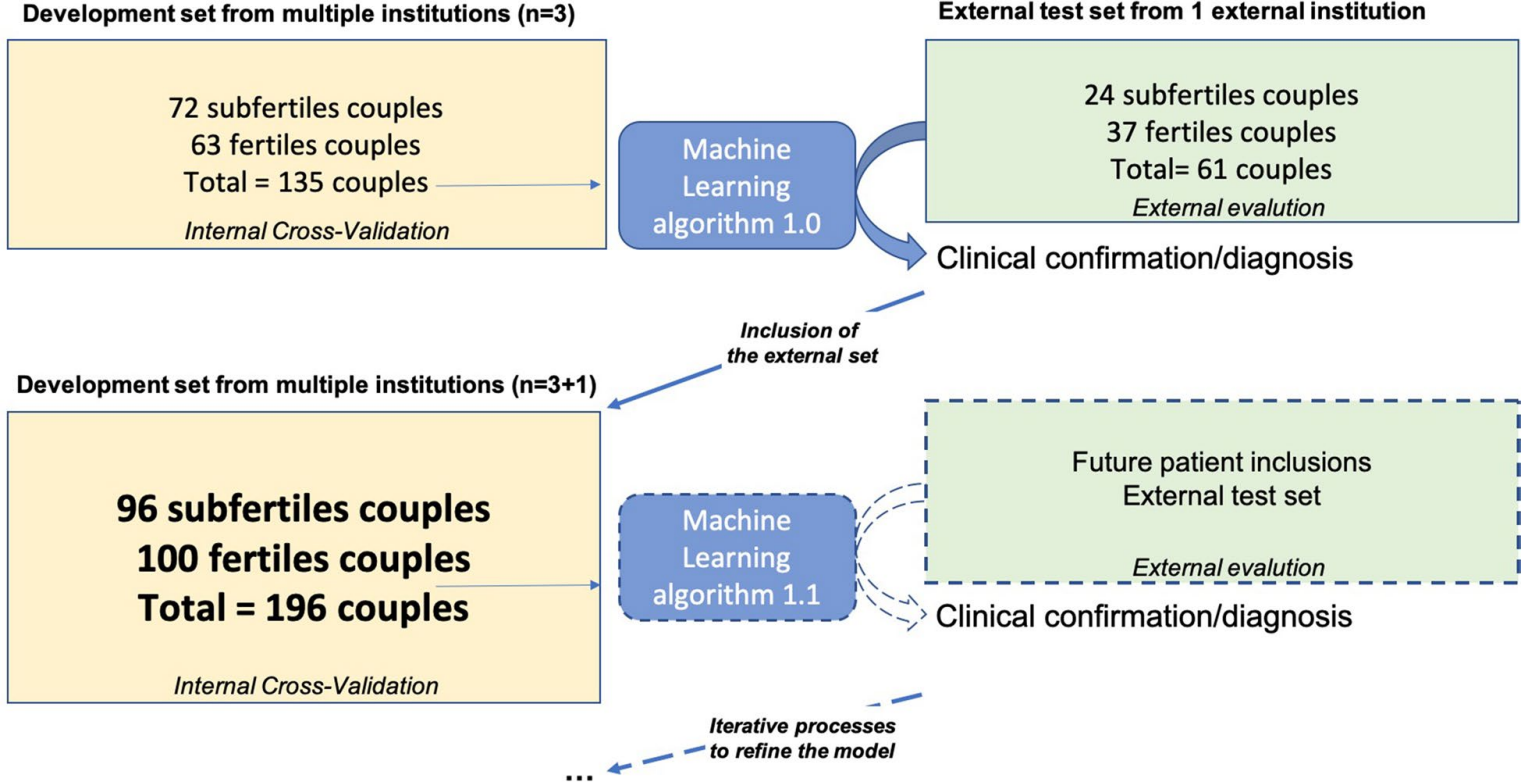
Data flow diagram for the study: model training iteration. The model was trained and tuned after variable selection on a development set originating from patients of mutiple specialized centers. Firstly the model was internaly evaluated using cross-validation. Then, a test set of couples originating from a different institution was used for an external validation. The model predictions were matched with the expected clinical output. Model training iteration (i.e. introduced externally) process was eventually performed to build a refined model with the selected features.

As described in Fig. 1, the OPLS model was evaluated for the goodness of fit (R^2^) and the capability to predict using an internal cross-validation (Q^2^)^32^. R2 and Q2 values are between 0 indicating a poor fit or poor capability to predict and 1 (highest fit or prediction by the model). An independent data set of 31 couples (*i.e. from the* CCH institution) was used to evaluate the model performance (ie. accuracy) by external validation.

In order to reduce the dimensionality of our system and to generate a more “user-friendly” model, the least relevant variables were eliminated in the model using their Variable Importance for Projection (VIP).

### Ethical approval

The ethics committee (“Comité de Protection des Personnes”, Paris France) approved the study. ALIFERT study (national biomedical research P071224/AOM 08,180:NEudra CT 2009-A00256-51/clinical trials NCT01093378). All the participants signed a written informed consent.

## Results

### Population description

Idiopathic infertile couples men and women showed significant differences in the anthropometric and biological/metabolic parameters compared to fertile couples. For example, BMI, waist circumference, visceral fat, and glycaemia, were higher in infertile men and women (Supplementary Table 1a, 1b) but HDL lower.

Out of 13 micronutrients and vitamins with an antioxidative activity, retinol, alpha-carotene, lutein, and beta-carotene levels were lower in infertile men compared to fertile (Supplementary Table 1c). In infertile women, alpha-carotene, beta-carotene, lycopene, and lutein levels were also lower than in fertile (Supplementary Table 1c). Following the usual recommendations, infertile women are supplemented with folate during the preconception period^33^, which explained why their plasma folate levels were higher compared to fertile. The 2 groups were comparable in terms of steroids except for testosterone lower in infertile men (Supplementary Table 1d).

### Overview of the ALIFERT dataset with principal component analysis

A principal component analysis (PCA) was first performed to reduce the large variety of biological differences along the prominent variation trends (Supplementary Fig. 1). A PCA showing the scores of the men, women and couples (respectively A, B and C) projected on the first factorial plan (t1, t2) does not showed the separation between fertile and infertile groups. The percentages of explained variance along the two most prominent components (*ie* the two most influential combinations of variables) were < 0.5 (R^2^ men = 0.264, R^2^ women = 0.307, R^2^ couples = 0.192), which suggested weak correlations between the variables. The score plots for men and couples showed an outlier (the patient (man) with BMI 45, was above average by more than 8 standard deviations). The corresponding couple was excluded from the further model calculations.

### Relevance of the couple approach in the model versus single men or women

In order to test the advantage of a couple-based approach, OPLS models were separately calculated from men, women and the merged data from the two matched genders (*i.e.* the couple-based approach) (Supplementary Fig. 2A–C). The discrimination of the alternative fertility status is clearly revealed by the separation of red and green (fertile) symbols. The overlapping scores between the discriminated fertility status was only 7 subfertile and 4 fertile couples out of 135 observations from the development set.

**Figure 2:**
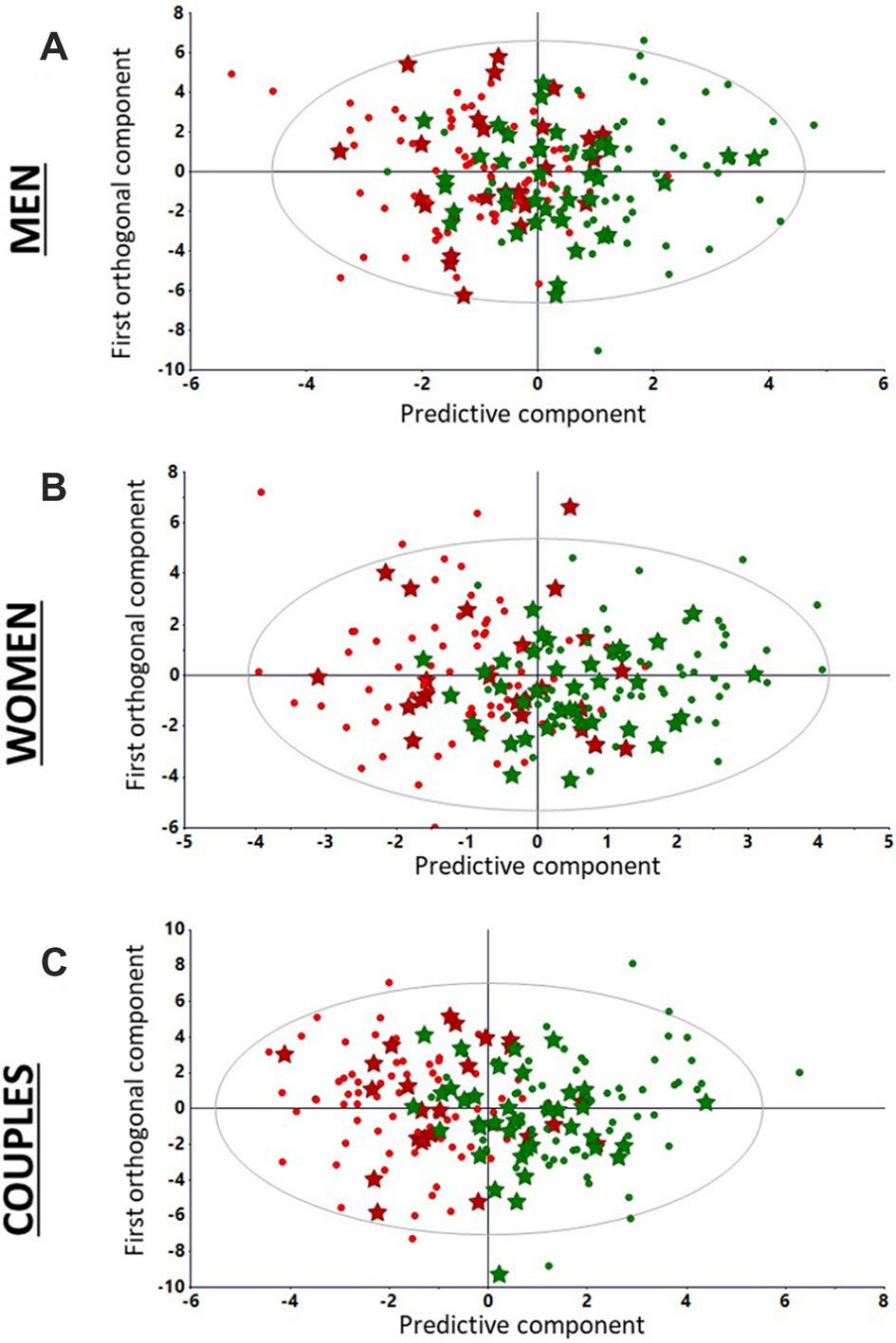
Orthogonal partial least squares discriminant analysis models (training and evaluation) calculated from men, women and couples development sets. (**A**) External evaluation of men fertility status: labelled external test set observations (fertile and infertile as green and red stars, respectively) are projected on the score plots generated from fertile (green dots) and infertile (red dots) men (135 × 50) set. R^2^ (fit) and Q^2^ (prediction capability) are 0.471 and 0.322, respectively. (**B**) External evaluation of women fertility status: labelled external test set observations (fertile and infertile as green and red stars, respectively) are projected on the score plots generated from fertile (green dots) and infertile (red dots) women (135 × 30) development set. R^2^ and Q^2^ were 0.560 and 0.462. (**C**) External evaluation of couples: labelled external test set observations (fertile and infertile as green and red stars, respectively) are projected on the score plots generated from fertile (green dots) and infertile (red dots) couples (135 × 80) development set. R^2^ and Q^2^ were 0.624 and 0.487. Accuracy scores are 0.590, 0.574 and 0.688 for models based on men, women and couples data, respectively.

Each of the three models described previously was tested on the independent validation set: the corresponding patients were plotted (black stars) regardless of their fertility (Supplementary Fig. 2–F) and secondly, their symbols were colored according to their fertile (green stars) or infertile status (red stars) (Fig. 2A–C). When the predicted status of the validation observations as based on the left- or right-quadrants position in the score plots are checked out, the model accuracy can be estimated to 59.0%, 57.4%, and 68.8% for the men, women, and couple-based models, respectively. Accordingly, the couple-based data set led to a more accurate and powerful model than the separate men and women models. We trained and evaluated 4 other machine-learning models (Support vector machine, Nearest Neighbors Classifier, Decision Tree and logistic regression) to confirm the discriminative power of the data, independently of the chosen method : as reported in supplemental Table 2, their performances were either worse or equivalent with OPLS-DA. These results confirmed the decision to adopt the OPLS-DA algorithm.

The Variable Importance for the Projection (VIP) histogram corresponding to the model summarized the relative influence of the 80 variables (Supplementary Fig. 3). In order to reduce the number of non-influential features regarding the discrimination of the fertility status, to limit the colinearity between redundant variables, to reduce the “noise” created the variance of less discrimant variables and to create a more parcimonius “user-friendly” model, we have proceeded to an iterative selection of the features as a function of the VIP (the higher the VIP, the more discriminatory the variable between the fertile/infertile groups).

Therefore, we successively selected features with a VIP score > 1 to obtain 24- (intermediate) and then 13-feature reductionist models.

### Refined models after selection of the fertility discriminating variables

**Figure 3:**
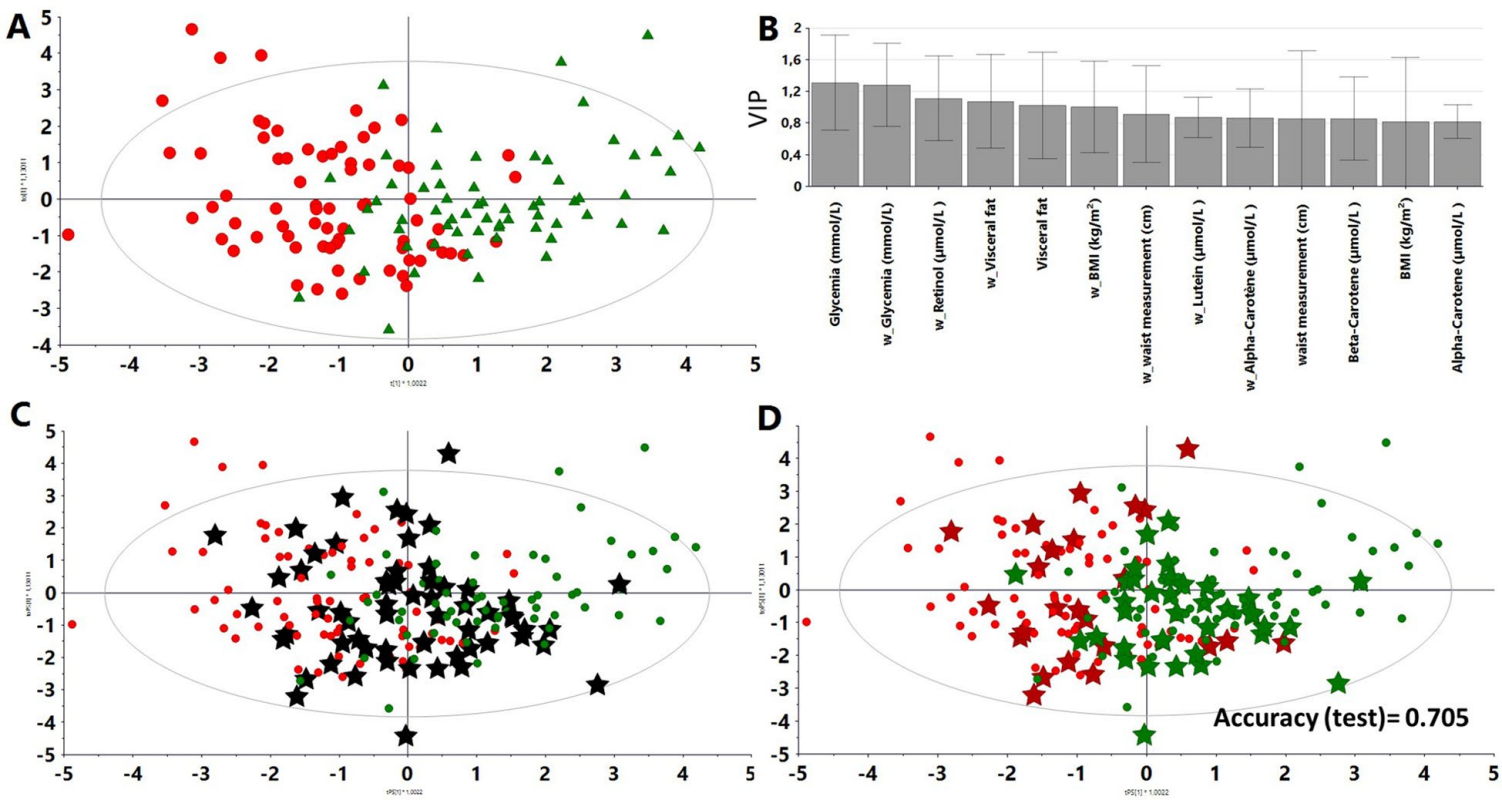
Supervised Orthogonal partial least squares discriminant (OPLS-DA) model with 13 variables selected from the 24 variables model with variable importance for projections (VIP) > 1: reductionist model. (**A**) Score plot generated from fertile (labeled as green triangles) and infertile couples (labeled as red dots). R^2^ and Q^2^ were 0.467 and 0.410, respectively. Accuracy (internal validation) = 0.838 and calculated on the test set = 0.705. (**B**) VIP (Variable Importance for the Projection) the plot summarized the influence of the 13 variables on the model. (**C**) Score plot reductionist model from the merged development and test sets (61 couples) (black stars). (**D**) Score plot reductionist model from the combined development and test sets. Scores are labeled according to their fertility status (green or red).

The iterative procedure has consisted to suppress in a stepwise maner the less influential variables with weak VIP and to calculate the accuracy of the resulting discriminant model for the couple fertility status prediction in the validation set. An intermediate model was built with 24 variables, of which the score plot exhibited a segregation between infertile and fertile groups (Supplementary Fig. 4A). The summary of the variable importance of the projection (VIP) histogram was calculated for the 24 variables (Supplementary Fig. 4B). Following iterative variable reduction/model validation, a discriminant model was calculated with 13 variables (Fig. 3A), of which the relative importance in the projection (VIP) was shown (Fig. 3B).

This model was then tested on the independent external test set: the corresponding couples were plotted (black stars) regardless of their label (Fig. 3C). Secondly, these couples were colored according to their fertile (green stars) or infertile (red stars) status (Fig. 3D). The accuracy score on the independent test set was 73.8%, and was therefore superior to that of the full-feature development set (68.8%). Based on these criteria, the model comprised of 13 variables was judged the most suitable to classify couples.

### High contribution of anthropometric, metabolic, and antioxidative parameters

Glycaemia, for both women and men, appeared to be the most important variable for scoring fertility. Circulating retinol also played a significant role as did to a lesser extent, the other antioxidative species such as lutein, beta-carotene, and alpha-carotene exhibiting a high VIP score.

Variations of anthropometric parameters such visceral fat, body mass index, and waist measurement in both women and men were among the most significant parameters in the model. Surprisingly steroid hormones and various antioxidative species, including lutein and selenium, which showed less relevance in the intermediate discriminant analysis with 24 variables were excluded from the refined model.

## Discussion

In this study, we proposed a proof of concept of a machine-learning model that classify fertile and infertile couples according to anthropometric, antioxidative, and metabolic signatures. Besides of thoroughly documented causes of infertility such as genetic, congenital, and acquired defects, increasing interest should also be focused on the environmental and lifestyle factors^21^. Indeed, addictions to tobacco, alcohol, and drugs, as well as diet, physical activity, obesity, metabolic disorders, sleep, and stress, also compromise male and female fertility^5,18,34–37^. Interestingly, the impact of these factors is reversible if apropriate corrective measures are taken. These risk factors are commonly studied separatly, while their effects may be cumulative in combination^38^. Therefore, it seemed helpful to build a model including the several lifestyle-related factors as well as the two members of the couple to quantitatively predict the infertility degrees. Furthermore, this model could facilitate and refine the medical decision-making process on the basis of a limited number of variables in the complex context of idiopathic infertility.

With the present model, a couple is considered a single entity rather than as two independent individuals. Our validation on an independent cohort showed that: (i) the couple-modeling approach was indeed more discriminant than a model in which men’s and women’s are considered separately; (ii) the most important variables for the projection were related to metabolic disorders, oxidative status, and antropometry; and (iii) after reduction of our system dimension (from 80 down to 13 variables), we proposed a new algorithm to discriminate fertile vs. infertile patients. Among the 13 variables of interest retained at the end of the model, anthropometric, antioxidative, and metabolic parameters are the main variables that can correlate with idiopathic infertility. Elevated blood glucose, lowered HDL cholesterol, antropometric parameters related to abdominal obesity (but no blood pressure) are suggestive of metabolic syndrome and discriminating of both men and women fertility status. The oxidative status is also consistent with the prolonged mild inflammation associated with the metabolic syndrome and importantly alpha-carotene and beta-carotene in men, as well as alpha-carotene, retinol, and lutein in women, are included in the refined model comprised of the most influential variables. These carotenoids have antioxidant actions and are present associated in a variety of fresh fruits and vegetables abundant in an appropriate diet (alpha-carotene, beta-carotene, and lutein) or in foods of animal origin (retinol).

Therefore, changing pre-conception nutritional and lifestyle factors should be considered a first-choice for unexplained infertility^39^. We observed a small overlap between the scores of fertile and infertile couples—and assume that patients will benefit of improvements to lifestyle factors to obtain live birth^16^. Prospective interventional studies are now needed to support the model and determine how lifestyle change the model calculated scores and correlate with the chances of pregnancy.

The definition of idiopathic infertility being related to the results of a standard protocol these observations lead potentially to a renewal of idiopathic infertility definition by including lifestyle parameters.

The reductionist model built from 135 couples has already shown its predictive capacity within an external cohort with a slight overfitting (83.8% on the training set vs. 73.8% on the test set). In addition, upon increasing the number of couples (from 136 to 196), a moderate decrease in accuracy on the training set (83.8–80.3%) was observed, likely reflecting an overfitting reduction. Afterwards, the final machine-learning model that is trained using the entirety of the data set (196 couples) will require further validation using an independent data set. Furthermore, this study focused only on couples with idiopathic primary infertility.

## Conclusion

The proposed model is a multivariate supervised statistical model built from data drawn from infertile men and women who are considered as a unique couple in observation. The couple approach yielded a significant gain in discriminatory power compared to models in which analysis of men or women are implemented separately.

The proposed model could find its place in the standard care management of couples with parental project. This approach is interesting since personalized lifestyle intervention should be considered as a first-choice treatment for idiopathic infertility and should be systematicaly suggested before ART. Prospective interventional studies will be needed to test the hypothesis demonstrated by this algorithm and to validate this model for clinical use. Moreover, it would also be interesting to assess whether this model would be effective in tracking the efficinecy of potential lifestyle modifications.

## Supplementary Information

Supplementary Information.

## Abbreviations

BMI: Body mass index
CI: Confidence interval
PCA: Principal component analysis
OPLS-DA: Orthogonal partial least squares discriminant analysis
ART: Assisted reproductive technology
IUI: Intra-uterine insemination
IVF: In vitro fertilization
VIP: Variable Importance for the projection
