# Supplementary material for: Proof of concept and development of a couple-based machine learning model to stratify infertile patients with idiopathic infertility
Source: Sci Rep. 2021 Dec 14;11:24003. doi: 10.1038/s41598-021-03165-3 (PMC8671584; doi:10.1038/s41598-021-03165-3)
Supplement: Supplementary file 1 — Supplementary Information. [file 41598_2021_3165_MOESM1_ESM.docx]

Supplemental materials and methods:

**Orthogonal Partial Least Squares:**

Partial Least Squares (PLS) regression is a multivariate regression method commonly used in chemometrics, a field of using advanced statistical and machine learning methods in analytical chemistry applications. One great advantage of this approach is its capability to deal with multicollinearity in application systems such as multivariate calibration and process analytical technology, with inverted matrix (i.e. fewer observations than variables).

The general underlying model of multivariate PLS is

X = TP^T^ + E

Y = UQ^T^ + F

where X is an n x m matrix of predictors, Y is an n x p matrix of responses; T and U are n x l matrices that are, respectively, projections of X (the X score, component or factor matrix) and projections of Y (the Y scores); P and Q are, respectively, m x l and p x l orthogonal loading matrices; and matrices E and F are the error terms, assumed to be independent and identically distributed random normal variables. The decompositions of X and Y are made to maximize the covariance between T and U.

O-PLS is an extension of the original PLS regression. O-PLS is a supervised multivariate data projection method used to relate a set of predictor variables (X= bioclinical signature) to one or more responses (Y = fertility status). This approach attempts to extract the maximum information reflecting the variation in the data set, while assuming the existence of a small subset of hidden variables in the X-data to predict the response variables. These subsets are formally called latent variables (or LVs) based on the same concept as Principal Component Analysis (PCA). The O-PLS method, uses orthogonal signal correction to maximize the explained covariance on the first LV, while the remaining LVs capture variance in the predictors which is orthogonal, i.e. statistically uncorrelated to the response variables.

|  | **Subfertiles (n = 96)** | **Fertiles (n = 100)** |  | ***p-value*** |
| --- | --- | --- | --- | --- |
| ***baseline parameters*** |  |  |  |  |
| Age (years) | 33.23 [32.17, 34.28] | 34.43 [33.67, 35.19] |  | ** |
| weight (kg) | 81.38 [78.63, 84.14] | 76.21 [74.23, 78.19] |  | ** |
| height (cm) | 177.82 [176.56, 179.09] | 178.32 [177.04, 179.60] |  | * |
| BMI (kg/m²) | 25.68 [24.94, 26.42] | 23.94 [23.42, 24.46] |  | *** |
| waist measurement (cm) | 91.06 [89.07, 93.06] | 86.14 [84.67, 87.62] |  | *** |
| hip measurement (cm) | 92.81 [91.05, 94.57] | 88.55 [87.17, 89.94] |  | *** |
| Visceral fat | 6.53 [5.81, 7.25] | 4.07 [3.64, 4.50] |  | *** |
| Blood_pressure_Systolic(mmHg) | 126.18 [123.82, 128.53] | 126.22 [123.95, 128.50] |  | * |
| Blood_pressure_Diastolic(mmHg) | 80.89 [78.89, 82.89] | 80.41 [78.54, 82.27] |  | * |
| CO_exp (ppm) | 5.76 [4.73, 6.79] | 3.55 [3.09, 4.02] |  | *** |
| w_age (year) | 31.03 [30.17, 31.89] | 32.16 [31.53, 32.79] |  | * |
| w_ weight (kg) | 65.43 [62.70, 68.16] | 59.71 [58.21, 61.20] |  | ** |
| w_height (cm) | 165.07 [163.93, 166.22] | 165.62 [164.63, 166.62] |  | * |
| w_BMI (kg/m²) | 24.08 [23.12, 25.04] | 21.87 [21.27, 22.48] |  | *** |
| w_waist measurement (cm) | 80.74 [78.52, 82.97] | 76.95 [75.49, 78.41] |  | ** |
| w_hip measurement (cm) | 89.27 [87.26, 91.28] | 84.81 [83.44, 86.19] |  | *** |
| w_Visceral fat | 3.63 [3.12, 4.14] | 2.26 [1.98, 2.53] |  | *** |
| w_Blood_pressure_Systolic(mmHg) | 112.71 [110.09, 115.33] | 111.44 [109.61, 113.27] |  | * |
| w_Blood_pressure_Diastolic(mmHg) | 76.35 [74.60, 78.09] | 74.95 [73.33, 76.56] |  | * |
| w_CO_exp (ppm) | 4.17 [3.38, 4.96] | 2.80 [2.50, 3.12] |  | *** |

**Supplementary Table 1a : Baseline characteristics of subfertile and fertiles couples. Data reported as mean and 95% CI (confidence interval).**

*Wilcoxon-Mann Whitney,  t-test or a Welch test* for means comparison between groups** : p-value* > 0.05, ** :  *p-value [0.001, 0.05] , *** : p-value < 0.001*

Note that the variables starting with « w » relate to data from women.

|  | **Subfertiles (n = 96)** | **Fertiles (n = 100)** |  | ***p-value*** |
| --- | --- | --- | --- | --- |
| ***Metabolic/biological parameters*** | |  |  |  |
| Glycemia (mmol/L) | 4.94 [4.81, 5.07] | 4.31 [4.13, 4.49] |  | *** |
| Cholesterol (mmol/L) | 5.21 [5.01, 5.42] | 5.20 [5.02, 5.38] |  | * |
| HDL (mmol/L) | 1.26 [1.18, 1.33] | 1.38 [1.32, 1.44] |  | ** |
| LDL (mmol/L) | 3.29 [3.09, 3.48] | 3.24 [3.06, 3.42] |  | * |
| Triglycerides (mmol/L) | 1.41 [1.22, 1.60] | 1.20 [1.06, 1.34] |  | * |
| Ferritin (µg/L) | 203.55 [177.09, 230.01] | 214.13 [194.27, 234.00] |  | * |
| Creatinine (µmol/L) | 81.54 [78.52, 84.55] | 85.65 [83.13, 88.17] |  | ** |
| vitamine D (ng/ml) | 22.97 [20.69, 25.25] | 20.78 [18.82, 22.74] |  | * |
| w_Glycemia (mmol/L) | 4.79 [4.66, 4.92] | 4.22 [4.05, 4.38] |  | *** |
| w_Cholesterol (mmol/L) | 4.80 [4.64, 4.97] | 4.90 [4.69, 5.11] |  | * |
| w_HDL (mmol/L) | 1.58 [1.50, 1.66] | 1.64 [1.56, 1.72] |  | * |
| w_LDL (mmol/L) | 2.83 [2.68, 2.97] | 2.81 [2.64, 2.99] |  | * |
| w_Triglycerides (mmol/L) | 0.84 [0.77, 0.92] | 1.63 [0.35, 2.92] |  | ** |
| w_Ferritin (µg/L) | 61.30 [49.23, 73.38] | 49.16 [40.93, 57.38] |  | * |
| w_Creatinine (µmol/L) | 65.11 [62.37, 67.85] | 67.07 [64.72, 69.43] |  | * |
| w_vitamine D (ng/ml) | 26.49 [23.84, 29.14] | 24.29 [21.70, 26.89] |  | * |

**Supplementary Table 1b : Metabolic and biological characteristics of subfertile couples. Data reported as mean and 95% CI (confidence interval).**

*Wilcoxon-Mann Whitney,  t-test or a Welch test* for means comparison between groups** : p-value* > 0.05, ** :  *p-value [0.001, 0.05] , *** : p-value < 0.001*

Note that the variables starting with « w » relate to data from women.

|  | **Subfertiles (n = 96)** | **Fertiles (n = 100)** |  | ***p-value*** |
| --- | --- | --- | --- | --- |
| ***Microelements and vitamins*** |  |  |  |  |
| folate (nmol/L) | 12.31 [10.89, 13.72] | 13.12 [11.86, 14.38] |  | * |
| cobalamin (pmol/L) | 298.27 [274.67, 321.86] | 310.68 [286.32, 335.05] |  | * |
| Retinol (µmol/L ) | 2.07 [1.97, 2.17] | 2.21 [2.12, 2.29] |  | ** |
| Alpha-Tocopherol (mmol/L) | 24.79 [23.31, 26.26] | 26.22 [25.26, 27.18] |  | ** |
| Zinc (µmol/L ) | 12.84 [12.47, 13.22] | 13.05 [12.66, 13.44] |  | * |
| Selenium (mmol/L) | 1.19 [1.15, 1.22] | 1.19 [1.16, 1.22] |  | * |
| Glutathione peroxidase | 385.91 [373.94, 397.87] | 399.58 [388.09, 411.06] |  | * |
| Ascorbic acid (mg/mL) | 42.64 [38.67, 46.61] | 40.48 [35.37, 45.58] |  | * |
| Alpha-Carotene (µmol/L ) | 0.14 [0.12, 0.16] | 0.20 [0.18, 0.23] |  | *** |
| Lycopene (µmol/L ) | 0.52 [0.46, 0.58] | 0.53 [0.49, 0.58] |  | * |
| Lutein (µmol/L ) | 0.31 [0.28, 0.34] | 0.39 [0.36, 0.41] |  | *** |
| Glutathione (µmol/L ) | 822.63 [780.41, 864.86] | 797.78 [756.02, 839.54] |  | * |
| Beta-Carotene (µmol/L ) | 0.45 [0.39, 0.50] | 0.64 [0.56, 0.72] |  | *** |
| w_Folate (nmol/L) | 19.52 [16.57, 22.46] | 14.73 [13.25, 16.20] |  | ** |
| w_cobalamin (pmol/L) | 381.91 [350.02, 413.80] | 333.54 [307.20, 359.89] |  | ** |
| w_Retinol (µmol/L ) | 1.56 [1.48, 1.63] | 1.94 [1.84, 2.03] |  | *** |
| w_Alpha-Tocophérol (mmol/L) | 23.54 [22.31, 24.77] | 25.24 [24.12, 26.35] |  | * |
| w_Zinc (µmol/L ) | 12.08 [11.74, 12.42] | 12.08 [11.73, 12.44] |  | * |
| w_Selenium (mmol/L) | 1.09 [1.06, 1.12] | 1.15 [1.12, 1.17] |  | ** |
| w_Vitamine C (mg/mL) | 54.31 [50.00, 58.62] | 42.75 [36.58, 48.92] |  | ** |
| w_Alpha-Carotène (µmol/L ) | 0.19 [0.16, 0.22] | 0.27 [0.23, 0.31] |  | *** |
| w_Lycopene (µmol/L ) | 0.45 [0.40, 0.50] | 0.51 [0.47, 0.55] |  | ** |
| w_Lutein (µmol/L ) | 0.29 [0.26, 0.32] | 0.38 [0.35, 0.41] |  | *** |
| w_Béta-Cryptoxanthine (µmol/L ) | 0.23 [0.19, 0.28] | 0.25 [0.21, 0.29] |  | * |
| w_Glutathione (µmol/L ) | 814.90 [769.82, 859.97] | 797.20 [759.43, 835.04] |  | * |
| w_Beta-Carotene (µmol/L ) | 0.68 [0.60, 0.76] | 0.83 [0.75, 0.92] |  | ** |

**Supplementary Table 1c : Microelements and vitamins characteristics of subfertile couples. Data reported as mean and 95% CI (confidence interval).**

*Wilcoxon-Mann Whitney,  t-test or a Welch test* for means comparison between groups** : p-value* > 0.05, ** :  *p-value [0.001, 0.05] , *** : p-value < 0.001*

Note that the variables starting with « w » relate to data from women.

|  | **Subfertiles (n = 96)** | **Fertiles (n = 100)** |  | ***p-value*** |
| --- | --- | --- | --- | --- |
| ***Hormones*** |  |  |  |  |
| ALDOSTERONE (pg/mL) | 74.72 [61.95, 87.49] | 82.86 [71.45, 94.26] |  | * |
| DHEA (ng/mL) | 7.02 [6.10, 7.94] | 6.04 [4.93, 7.15] |  | * |
| ANDROSTENEDIONE (ng/mL) | 0.93 [0.84, 1.03] | 0.93 [0.84, 1.02] |  | * |
| TESTOSTERONE (ng/mL) | 4.65 [4.25, 5.04] | 5.28 [4.85, 5.71] |  | ** |
| DHT (ng/mL) | 0.65 [0.59, 0.71] | 0.65 [0.59, 0.71] |  | * |
| Pregn (ng/mL) | 1.49 [1.18, 1.80] | 1.49 [1.31, 1.66] |  | * |
| 11 BOH4 (ng/mL) | 1.43 [1.25, 1.61] | 1.45 [1.30, 1.61] |  | * |
| 17OH Pregn (ng/mL) | 2.81 [2.27, 3.35] | 3.26 [2.16, 4.36] |  | * |
| PROGESTERONE (ng/mL) | 0.10 [0.06, 0.14] | 0.10 [0.08, 0.12] |  | * |
| 16OHP (ng/mL) | 0.20 [0.16, 0.24] | 0.22 [0.18, 0.26] |  | * |
| 21DB (pg/mL) | 1.36 [-1.08, 3.79] | 3.67 [2.27, 5.07] |  | ** |
| DOC (pg/mL) | 40.81 [32.33, 49.29] | 48.57 [36.05, 61.08] |  | * |
| 17OHP (ng/mL) | 1.04 [0.85, 1.23] | 1.04 [0.92, 1.16] |  | * |
| 21DF (ng/mL) | 0.03 [0.02, 0.05] | 0.03 [0.02, 0.05] |  | * |
| CORTICOSTERONE (ng/mL) | 3.79 [2.94, 4.65] | 4.53 [3.29, 5.78] |  | * |
| 11DF (ng/mL) | 0.35 [0.27, 0.42] | 0.38 [0.30, 0.47] |  | * |
| CORTISONE (ng/mL) | 19.87 [18.71, 21.03] | 20.11 [18.90, 21.33] |  | * |
| CORTISOL (ng/mL) | 101.70 [91.05, 112.35] | 105.56 [93.98, 117.13] |  | * |
| AMH (ng/ml) | 9.56 [8.42, 10.70] | 9.40 [8.42, 10.38] |  | * |
| w_AMH (ng/ml) | 3.04 [2.49, 3.59] | 3.31 [2.69, 3.93] |  | * |

**Supplementary Table 1d : Hormonal characteristics of subfertile couples. Data reported as mean and 95% CI (confidence interval).**

*Wilcoxon-Mann Whitney,  t-test or a Welch test* for means comparison between groups** : p-value* > 0.05, ** :  *p-value [0.001, 0.05] , *** : p-value < 0.001*

Note that the variables starting with « w » relate to data from women.

|  | Accuracy | Sensibility | Specificity |
| --- | --- | --- | --- |
| Logistic regression | 62.3 | 54.2 | 67.6 |
| Decision Tree | 62.3 | 70.8 | 56.8 |
| K-Neighbors classifier | 57.4 | 79.2 | 43.2 |
| Support vector machine | 62.3 | 79.2 | 51.3 |
| OPLS-DA | 68.8 | 83.3 | 67.7 |

**Supplementary Table 2 : Algorithm’s performances of 5 machine learning models from couples development sets (full features) and score from test set. Comparison of several evaluation metrics (Accuracy, sensibility and specificity).**


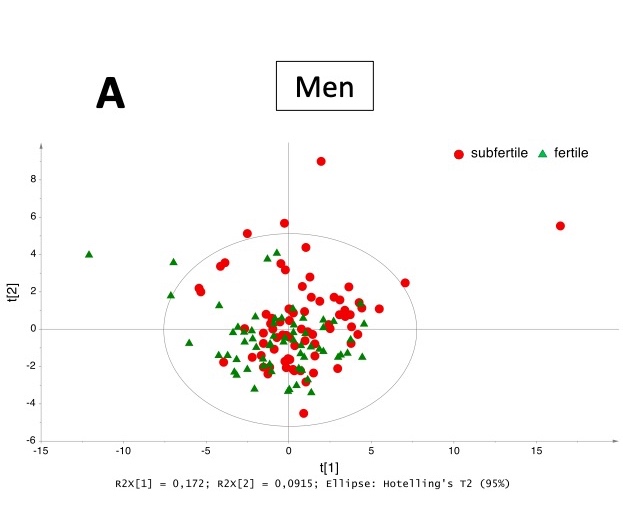

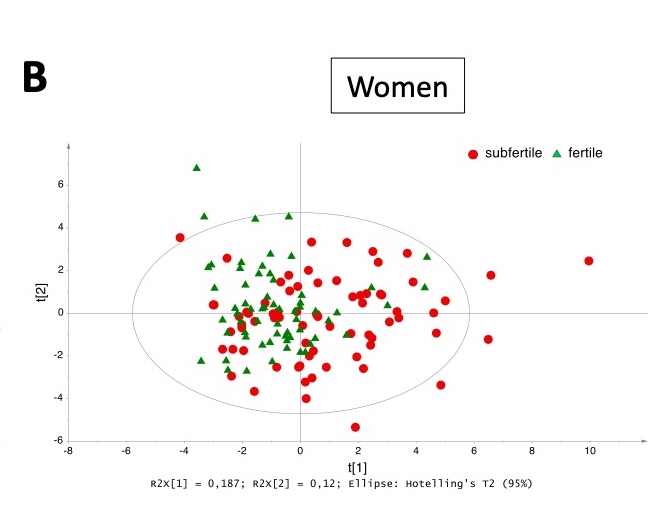

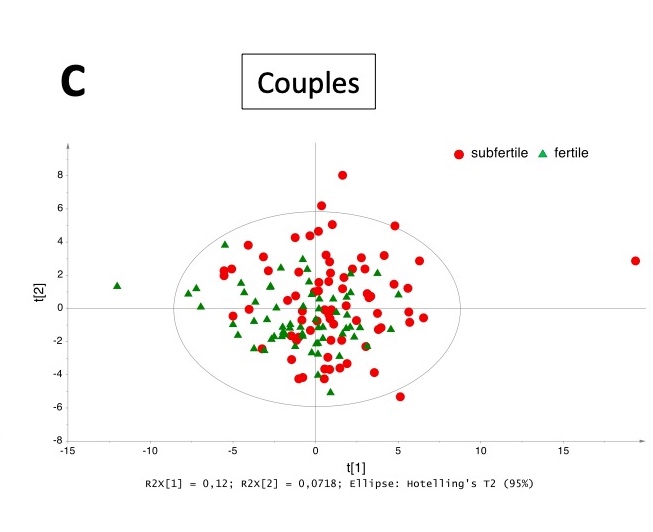


**Supplementary Figure 1 : Principal Component Analysis Score Plots of fertile and subfertile groups based on men (A), women (B) and couples (C) from development set.** The 2 principal components (t1 and t2) were presented for each score plot. Data set matrix dimension (Observations x Variables). A. Score plot mens (136x50). B. Score plot womens (136x30). C. Score plot mens + womens (couples) (136x80). R^2^ were 0.264, 0.307 and 0.192 for men, women and couples respectively. The score plots for men and couples showed one outlier among the observations : this couple outlier was excluded from the model.

**
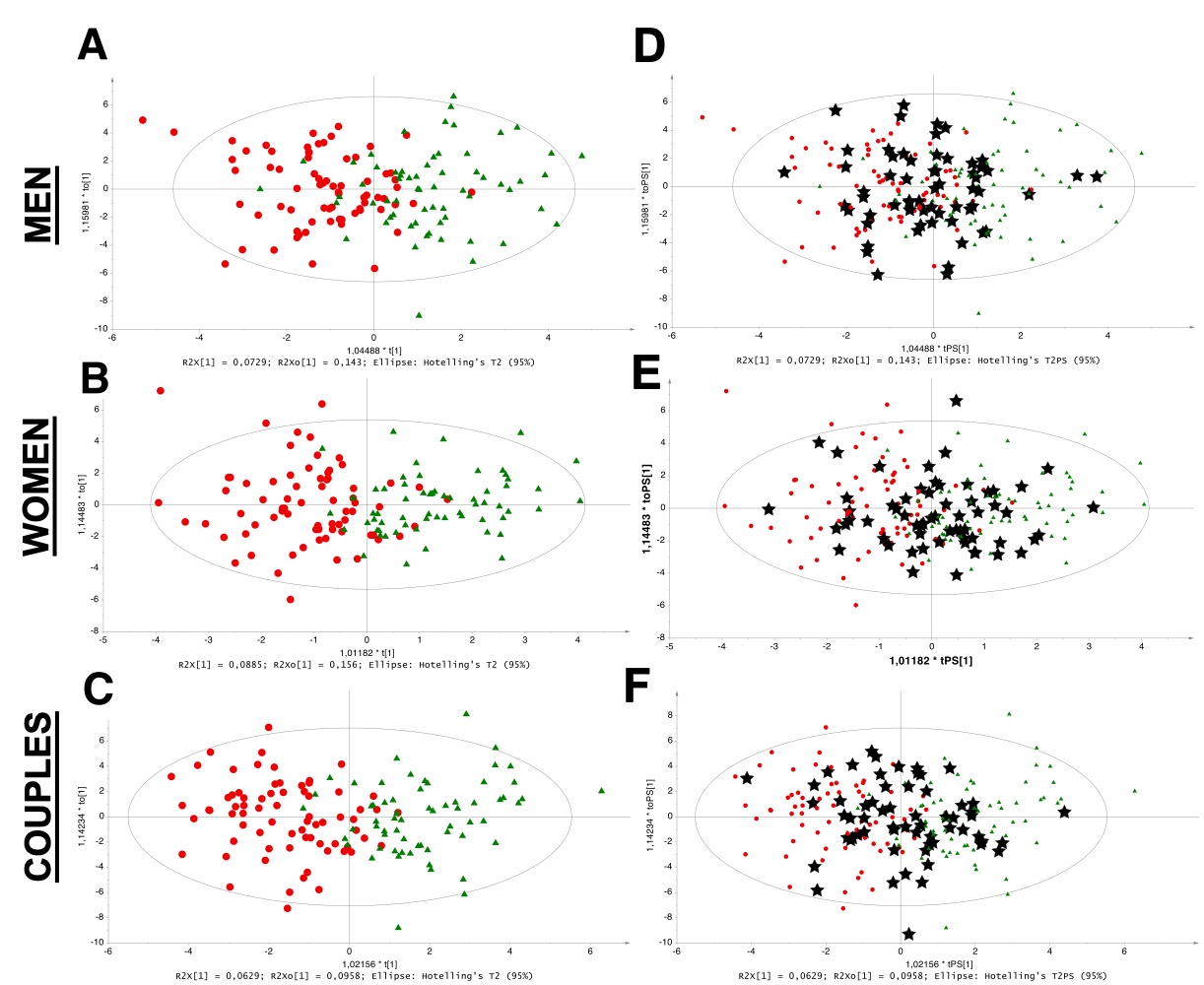
**

**Supplementary Figure 2 : Orthogonal partial least squares Discriminant Analysis models (building and external evaluation) from either men, women and couples development sets (full features) : score plot and unlabelled test set.**

(A,B,C) : Score Scatter plots generated from fertile (green triangles) and subfertile (red dots) men (135x50), women (135x30) and couples (135x80) development set. R^2^ and Q^2^ were 0.471/0.322, 0.560/0.462, and 0.624/0.487 for men, women and couples respectively.

(D,E,F) : Unlabelled external test set observations (black stars n=61) prior to clinical matching, projected on the score Scatter plots generated from fertile (green dots) and subfertile (red dots) men (135x50), women (135x30) and couples (135x80) development set.


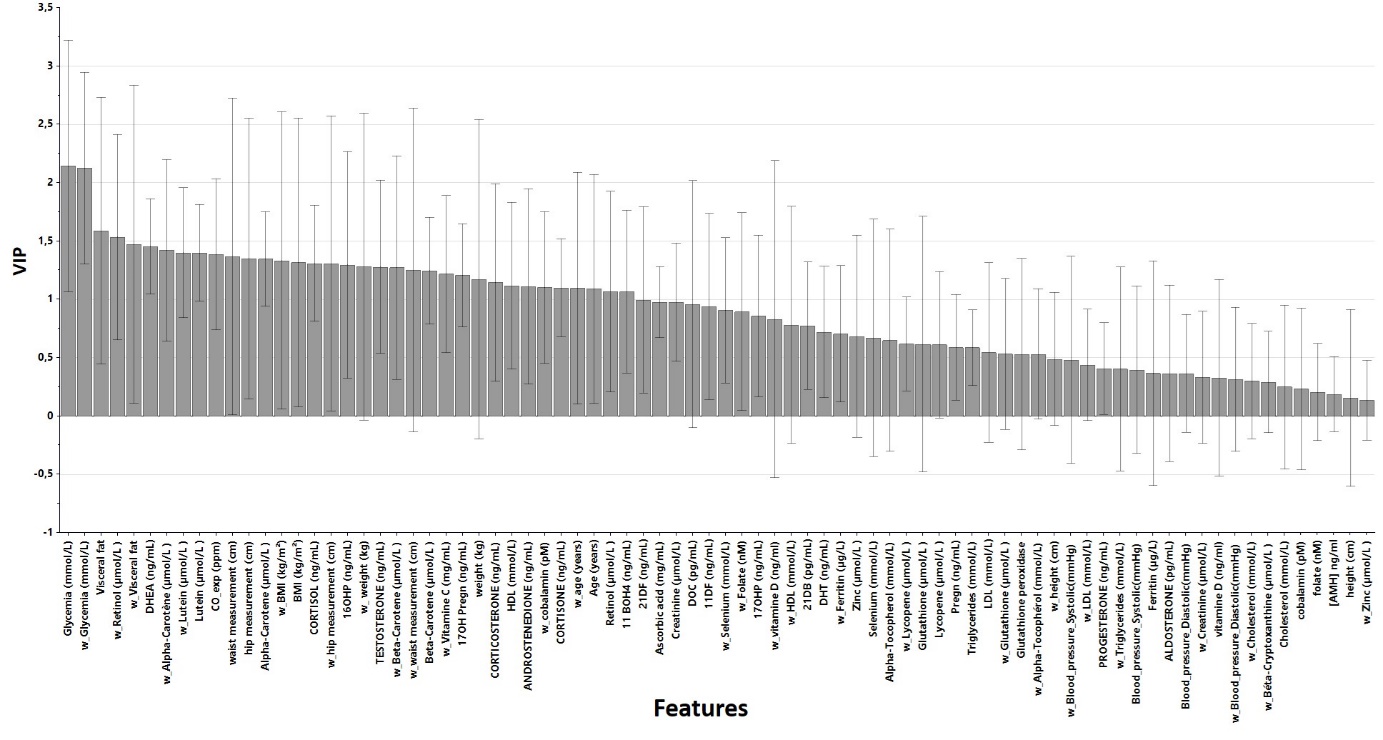


**Supplementary Figure 3 : Variable Importance for the projection (VIP) plot for development set couples from the OPLS-DA : 135 couples and 80 features.**

**
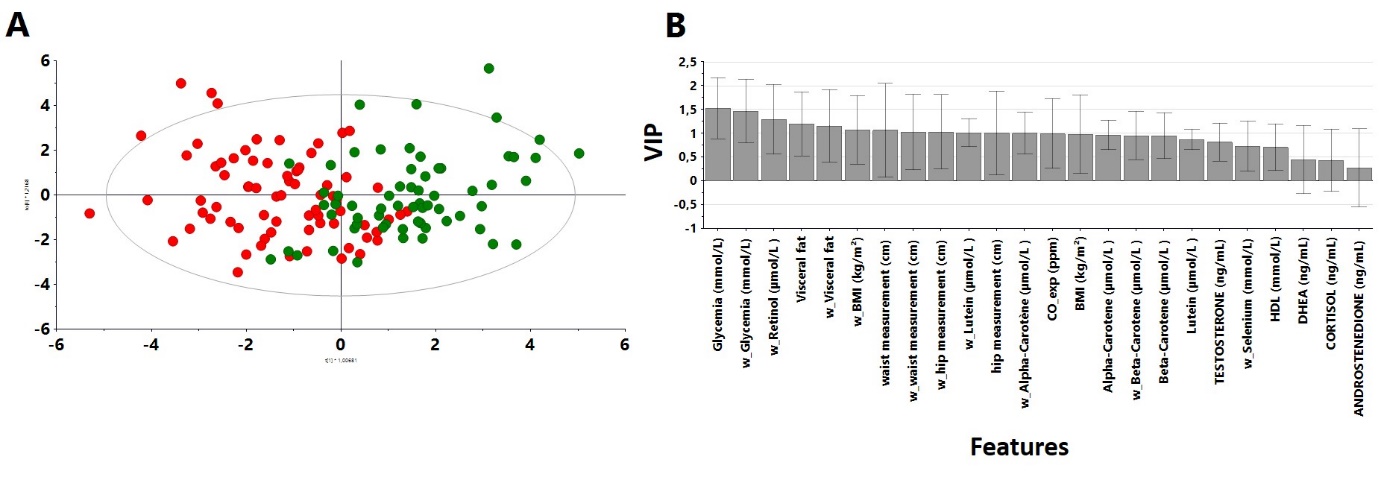
**

**Supplementary Figure 4 : Supervised Orthogonal partial least squares Discriminant analysis (OPLS-DA) with 24 variables selected from the full development set model (80 variables) with VIP > 1.** A. Score Scatter Plot R^2^ and Q^2^ were 0.534 and 0.472 respectively. Mean accuracy score = 0.890 B. VIP (Variable Importance for the Projection) plot summarized the importance of the 24 variables both to explain X and to correlate to Y.

The summary of the variable importance of the projection (VIP) histogram was calculated for 24 variables comprising 3 main categories (Supplemental figure 3B): The antioxidative species such as Retinol Alpha- and Beta-Carotene or lutein appeared to be important for the discrimination. Variations of anthropometric parameters such body mass index, hip measurement and visceral fat measured on both women and men were also among the most significant parameters for the model. The contribution of biological variables such as glycemia (for women and men) and steroid hormones for men were also part of the list in term importance for the projection. In addition, some variables still showed a VIP below 0.5 (testosterone, dehydroepiandrosterone (DHEA), cortisol, and 16-OHP), which indicated poor relevance for the projection. They could be removed in a more compact model with fewer variables.
